# Epidemiology and outcome of articular complications in adult onset still's disease

**DOI:** 10.11604/pamj.2015.22.77.6366

**Published:** 2015-09-30

**Authors:** Madiha Mahfoudhi, Rafik Shimi, Sami Turki, Adel Kheder

**Affiliations:** 1 Internal Medicine A Department, Charles Nicolle Hospital, Tunis, Tunisia

**Keywords:** Adult onset still′s disease, arthritis, fever, corticosteroids, biotherapy, prognosis

## Abstract

The adult onset Still's disease is a rare inflammatory pathology of unknown pathogeny. The clinical features are variable. The diagnosis is difficult since exclusion of infectious, systemic and tumoral pathologies should be done. The articular complications are frequent and can be revelatory of this pathology. The articular prognosis depends on the diagnosis delay and the treatment efficiency. Our study aims to analyze different aspects of articular manifestations complicating adult onset Still disease to define epidemiological, clinical and evolving characteristics of these complications. It was a cross-sectional study concerning 18 cases of adult onset Still disease diagnosed from 1990 to 2014 in the internal medicine A department of Charles Nicolle Hospital in Tunis, meeting Yamaguchi criteria. We identified clinical, radiological, evolving and therapeutic profile of the articular manifestations occurred in these patients. There were 11 women and 7 men. The average age was 27 years. The arthralgias were reported in all cases; while, the arthritis interested thirteen patients. A hand deformation was found in four patients. A wrist ankylosis was noted in one case and a flexion elbow in one patient. The Standard articular radiographs were normal in ten cases. The treatment associated essentially non-steroidal anti-inflammatory and/or corticosteroids and/or methotrexate. Concerning the evolving profile, the monocyclic form was present in 25% of the cases, the intermittent form in 40% and the chronic articular form in 35% of our patients. The adult onset Still's disease is rare and heterogeneous. The articular disturbances are frequent and have various outcomes.

## Introduction

Adult onset Still's disease (AOSD) is a rare inflammatory disease of unknown etiology and pathogenesis. It is a highly heterogeneous disease entity both in clinical expression and in its outcome profile. In this disease, joint damage is frequent and unpredictable. Arthralgias are among the major criteria for diagnostic classifications Yamaguchi [1] and Fautrel [2]. The the management of joint manifestations is complex. This is primarily related to diagnostic difficulties in absence of specific signs and no codified therapeutic strategies.

## Methods

Our work is a study of 18 cases with review of the literature. This is a coss-sectional single-center study which consist of analyzing the epidemiological, clinical, radiological, evolving and therapeutic profile of patients with joint disease complicating AOSD, who were followed between 1990 and 2014 in the department of internal medicine A hospital Charles Nicolle. Then a review of many published series concerning articular disturbances in AOSD was performed. The AOSD, considered as a diagnosis of exclusion, was selected by the criteria of Yamaguchi, after eliminating connective vasculitis, infectious diseases and cancers. Through this series and a literature review, our objective was to analyze the main epidemiological, clinical and radiological characteristics of articular complications in AOSD, and to discuss therapeutic modalities for the different clinical presentations.

## Results

We collected 18 cases of AOSD who presented aricular manifestations due to this disease. The average age at diagnosis was 27 years with extremes ranging from age 18 to 58 years. Our patients were distributed in 11 women and 7 men. The sex ratio was 0.63. The mean diagnosis delay was 11 months. Extremes of this delay ranged between 1 and 40 months with a median of 3 months. The total duration of all hospitalizations in our patient during their follow-up was between 6 and 152 days with a median of 42 days. The duration of follow-up in our service was between 10 days and 150 months (13 years) with a mean of 57 months. In our series, all patients had fever at first, with hectic aspect and moving between 38,5°C and 40°C. Arthralgia was the plaint of all our patients. They had inflammatory poly-arthralgia and variable topography: These aches touched the big joints (knees, ankles, shoulders, hips) and small joints (metacarpo-phalangeal and inter-phalangeal joints). However, arthritis was present in thirteen patients and was unilateral or bilateral manifesting in varied topography. Wrists and knees were the most affected (7 and 9 patients, respectively). Hands deformity was found in four patients. It was respectively of a spindle deformation in one case and a curvature in three cases. An elbow flexion was observed in two patients. It was unilateral flexion in both cases. Ankylosis was noted in one patient and interested right wrist. Articular puncture was performed in two patients. Analysis of joint fluid was normal in the two cases. Synovial biopsy was performed in three patients. It showed an inflammatory synovitis in all patients. Joint radiographs of the affected joints were normal in ten patients. A decrease in bone mineralization was shown in five cases. A narrowing of the joint space was noted in three patients and interested elbow in the first patient, hands and sacroiliac in the second when he touched the L5-S1 in the third patient. A case of ankylosis of the right wrist and one case of bilateral hip disease have been reported. Therapeutic strategies adopted two therapeutic levels in our series: a first-line treatment (non-steroidal anti-inflammatory drugs (NSAIDs) and corticosteroids) and a second-line treatment (maintenance treatment). NSAIDs were used in 70% of our patients. They were ineffective as monotherapy. Their association with corticosteroids was more effective. Corticosteroids were widely used (in 95% of patients) and they had proved efficiency in 65% of patients who received prednisone in association to NSAIDs. Prednisone administrated in monotherapy was effective in 25% of patients. Methylprednisolone injected intravenously was effective in treating severe forms of the MSA. We used this treatment for a case of multi-organ involvement and two cases of fulminant hepatitis. There was a positive response to treatment in the three cases. Half of the patients received methotrexate (MTX). It was prescribed after the failure of corticosteroid therapy. The MTX-corticosteroid combination was very effective and had achieved a complete remission in 62% of patients who have benefited from this association. In the remaining patients, relapses were rare and occurred on an average interval months of 36 months, with 13 years interval in one case. The intolerance to MTX was only observed in one patient consisting of digestive disorders. In our series, no patient received biotherapy. The infiltration of corticosteroids had been indicated in three patients. One patient received osmic acid synoviorthesis and in another we proceeded with the implementation of a total hip replacement for a destructive hip disease. Evolutionarily, the moncyclic form was present in 25% of our patients, intermittent form in 40%, and chronic articular form in 35% of our patients.

## Discussion

Epidemiological data on the AOSD are very relative and incomplete. Currently, there is no consensus on the incidence and prevalence in different populations [3]. AOSD affects the majority of ethnic groups [3]. In Tunisia, the epidemiological data on the AOSD remain incomplete until this day, and scientific research on this subject has been exposed as a work on a series of patients or a medical doctorate thesis subject [4]. As part of our study, we were able to identify 18 patients suffering from AOSD whose diagnosis was made according to the criteria of Yamaguchi. Analysis of the literature showed that the series AOSD begins between 16 and 35 years in about 64% of patients with an average age between 21 years [5] and 38.19 years [6]. Our series noted a female predominance. The first description of the AOSD by Bywaters was derived from work on 14 women [7]. In most reported sightings through, there were a slight female predominance of the disease [5]. The diagnosis delay of AOSD in our study was comparable to that reported by the observations and series of cases; it was estimated at 5.4 months by Evensen KJ et al [8], 2 month by Fraisse TC et al [9], 7.32 months by Uppal SS et al [10] and 0.8 months by Crispín JC et al [11]. In Tunisian series, the average time to diagnosis was estimated at 6.8 months by Meddeb N et al [4]. The diagnosis of AOSD is still difficult. This diagnostic difficulty is intelligible in the epidemiological literature data. We waited in a case a 13-year period to confirm the diagnosis of MSA [12]. The duration of follow MSA is variable depending on the series and publications. This fact is explained by the difficulty of diagnosis, incomplete clinical picture at the beginning, and the unpredictable course of the disease [7–9]. Singh et al [13] evaluated this average to 19.14 months in a series of 14 cases. Riera E et al. [6] revealed an average follow-up period estimated of 112.85 months in an Italian series of 41 patients. The classic clinical trial of fever, arthralgia, skin rashes is a typical feature of the disease. However, the beginning of the MSA may be manifested by a fever that is associated only with arthritis in 25% of cases or only with a rash in 11% of cases reported in a series of literature [14, 15]. The exacerbation of joint pain is usually observed during fever spikes [15]. Our patients also had the same sequence of joint symptoms. In our series, all patients complained of joint pain while arthritis is diagnosed in 72.22% of patients. Our patients showed a variety of appearance and topography of joint manifestations and conform to the contributions of the literature. Arthritis can be initially mild, and transient oligoarticular form [16], but it can turn into a more serious form, destructive, symmetric and polyarticular over a period of several months [5].

Early in the disease, joint radiographs are normal and can't contribute to the diagnosis. They can sometimes show in the initial phase of the AOSD a joint swelling indicating the presence of synovitis or a discrete demineralization [15, 16]. Subsequently, we can reveal osteochondral lesions with joint destruction, especially in hips and knees. [15] About 41% of patients had an inter-articular space reduction of the wrist, carpal and carpometacarpal joints [16]. Joint destruction interests partcularly hips and knees in some cases and requires the establishment of a total prosthesis; non-erosive ankyloses of carpo-metacarpal and inter-carpal joints appear after a few years in AOSD [15]. The joint fluid had frequently inflammatory aspect. There are only a few observations of AOSD revealing mechanical fluid in the joints [15]. The synovial biopsy in the AOSD is useless according Pouchot et al [17]. When it was performed, it showed nonspecific acute synovitis which is comparable to that found in our series. Treatment of AOSD was essentially empirical. According to the literature, NSAIDs did not allow resolution of symptoms in 20% of cases. The efficiency of corticosteroids in the AOSD observations was spectacular in the majority of cases [5]. The dosages and duration of steroid therapy are not defined by consensus; they depended on the severity of the initial manifestations and their evolution under treatment. Iatrogenic complications attributed to corticotherapy were not uncommon. Methotrexate allowed in many cases the control of inflammatory activity in AOSD, with a corticosteroid sparing effect [5]. Contrary to MTX, other immunosuppressants used in the AOSD have not proven the effectiveness required according to published observations [3]. The treatment of severe forms and who have failed after methotrexate treatment has legitimized the use of new therapies, particularly biological therapy [18–20]. According to published observations and experiences of these new molecules, it was concluded that: Anakinra and tocilizumab appears to be more effective than anti-TNF-α (infliximab, etanercept, or adalimumab). Anti-TNF-α and anti-IL-6 seems more useful in chronic arthritis. The anti-IL-1 would be more effective in systemic manifestations [18, 19]. AOSD could engage both the functional and vital prognosis. Functional outcome was mainly due to joint damage, which could be erosive or destructive in chronic joint form and thereby leave sequelae in a third of patients.

## Conclusion

Still's disease in adults is a rare and ubiquitous inflammatory disease. The adoption of clinical and biological criteria for classification and the mastery of cardinal signs concerning joint complications may contribute to the diagnosis but they are still insufficient to assert definitively.
